# *MTHFR* 677C → T genotype modulates the effect of a 5-year supplementation with B-vitamins on homocysteine concentration: The SU.FOL.OM3 randomized controlled trial

**DOI:** 10.1371/journal.pone.0193352

**Published:** 2018-05-29

**Authors:** Leopold K. Fezeu, Veronique Ducros, Jean-Louis Guéant, Jean-Claude Guilland, Valentina A. Andreeva, Serge Hercberg, Pilar Galan

**Affiliations:** 1 Université Paris 13, Equipe de Recherche en Epidémiologie Nutritionnelle (EREN), Centre de Recherche en Epidémiologie et Statistiques, Inserm (U1153), Inra (U1125), Cnam, COMUE Sorbonne Paris Cité, Bobigny, France; 2 Département de Biochimie Pharmacologie et Toxicologie, UM Biochimie Nutritionnelle et Hormonale, Institut de Biologie et Pathologie, Centre Hospitalier Universitaire, Grenoble, France; 3 Inserm U724, Pathologies Cellulaire et Moléculaire en Nutrition, Faculté de Médecine, Université Henry Poincaré, Vandoeuvre lès Nancy, France; 4 *LPPCE*, Faculté de Médecine, Université de Bourgogne, Dijon, France; 5 Département de Santé Publique, Hôpital Avicenne, Bobigny, France; Griffith University - Gold Coast Campus, AUSTRALIA

## Abstract

**Aims:**

To study how *MTHFR* 677C→T genotype modulates the effect of supplementation with B-vitamins on total homocysteine (tHcy) and B-vitamin concentrations.

**Methods:**

2381 patients with a personal history of cardiovascular disease were randomly assigned to one of four groups: 1) B-vitamins alone (560 μg of 5-methyl-THF, 3 mg of vitamin B_6_ and 20 μg of vitamin B_12_), 2) n-3 fatty acids alone (600 mg of EPA and DHA in a 2:1 ratio), 3) B-vitamins and n-3 fatty acids, and 4) placebo. Participants were followed up for 4.7 years. At baseline and annually thereafter, biological parameters were assessed. Multivariate and linear mixed models were fit to study the interaction between B-vitamins and *MTHFR* genotype.

**Results:**

Among supplemented participants, concentrations of all three B-vitamins increased during the first year (all p<0.0001) across *MTHFR* genotype categories. tHcy decreased by 26.3% during the first year (p<0.0001), then steadily increased throughout the 5 years (p_trend_<0.001). However, at the end of follow-up, that increase was smaller among *TT* than among *CT* or *CC* subjects (p_interaction_<0.02). At baseline, the difference in tHcy concentrations between *TT* homozygous and *CC* homozygous subjects was 2.33 μmol/l (p<0.001). After 5 years, that difference was reduced to 1.06 μmol/l and remained statistically significant (p<0.001).

**Conclusion:**

Participants with the TT genotype exhibited a lower 5-year decrease in tHcy concentrations following a B-vitamin supplementation than did participants with the CC or CT genotype.

**Clinical trial registration:**

Current Controlled Trials # ISRCTN41926726.

## Background

Homocysteine (Hcy) is a sulfur-containing amino acid that plays a major role in methionine metabolism. Elevated plasma total homocysteine (tHcy) concentrations can be related to genetic defects, abnormal vitamin status, or both [1]. Methylenetetrahydrofolate reductase (MTHFR) converts 5,10-methylene tetrahydrofolate to 5-methyltetrahydrofolate (the main circulating form of folate) required for the conversion of Hcy to methionine; therefore, MTHFR plays a pivotal role in Hcy metabolism [2] by contributing to lowering its plasma values. A common mutation exists in the gene encoding the MTHFR enzyme. Individuals who have a *C-to-T* substitution at base 677 of the gene (amino acid change A222V) have lower enzyme activity, higher Hcy [3] and lower folate levels than do those without this mutation [4–7]. In addition to genetic defects, inadequate plasma concentrations of vitamin cofactors (e.g., vitamin B_6_, vitamin B_12_, and folate) play an important role in the regulation of plasma tHcy concentration [4, 8].

Elevated plasma values of tHcy have been related to chronic diseases, such as cardiovascular diseases (CVD) [9–12], osteoporotic fractures, end-stage renal disease, neurodegenerative diseases, migraine and neural tube defects[9–17]. In North America, folic acid fortification for the prevention of neural tube defects has been mandatory since 1998, resulting in a twofold increase in plasma folate values at the population level [18, 19]. Folic acid fortification is also practiced [18] in Chile, Argentina, Brazil, South Africa and Australia [20–22], but in New Zealand and in several West European countries, it has not been initiated, partly due to concerns regarding possible adverse effects on cancer incidence. [23, 24]

Small-scale studies have documented an effect of the *MTHFR 677* genotype on response to folic acid supplementation [25, 26]; other research has reported a reduction in disease risk related to a *MTHFR* 677 mutation through folic acid supplementation [27, 28]. Most of these studies were performed in populations with low folate levels. To our knowledge, except for the study by Crider et al. [29], which included only Chinese women of childbearing age, no population-based (including men and women) or large-scale, long-term, double-blind trials examining such associations have been reported.

We conducted post-hoc analyses of the “SUpplementation with FOLate, vitamins B_6_ and B_12_ and/or OMega-3 fatty acids” (SU.FOL.OM3) trial data to study the effect of supplementation with B-vitamins for 4.7 years on plasma concentrations of tHcy, folate, cobalamin and vitamin B_6,_ and to test whether there was an interaction between 677 C→ T mutation and the B-vitamin supplementation regarding change in tHcy concentration over time.

## Methods

### Study design

The SU.FOL.OM3 trial [30] was a multicenter, double-blind, placebo-controlled randomized trial with a factorial design, that evaluated the separate and combined effects of daily dietary dose supplementation with B-vitamins and n-3 polyunsaturated fatty acids on the secondary prevention of CVD. Briefly, 2,501 participants (1,987 men and 514 women) with a history of CVD (acute coronary or cerebral ischemic event occurring within 1 to 12 months prior to randomization) were eligible to participate. Inclusion took place between 1 February 2003 and 1 June 2007, and followed up ended on 1 July 2009. Using a two-by-two factorial design, subjects were randomly assigned to one of four groups: Group A: B-vitamins (5-methyl-THF [560 μg], vitamin B_6_ [pyridoxine hydrochloride, 3 mg] and vitamin B_12_ [cyanocobalamin, 20 μg]); Group B: n-3 fatty acids (600 mg of eicosapentaenoic acid (EPA) and docosahexaenoic acid (DPA) in a ratio of 2:1); Group C: both B vitamins and n-3 fatty acids; Group D: placebo (gelatin). All supplements were given as two capsules to be taken once daily. The median treatment/follow-up duration was 4.7 years.

The trial protocol [31] was approved by the ethics committee of the Paris-Cochin Hospital ("Comité consultatif pour la protection des personnes se prêtant à la recherche biomédicale," CCPPRB no. 1933) and the national data protection board ("Comité National de l’Informatique et des Libertés," CNIL no. 901230). Participants provided written informed consent. All analyses and data interpretation were independent of the organizations that funded this study.

### Measurements

**Fig 1:**
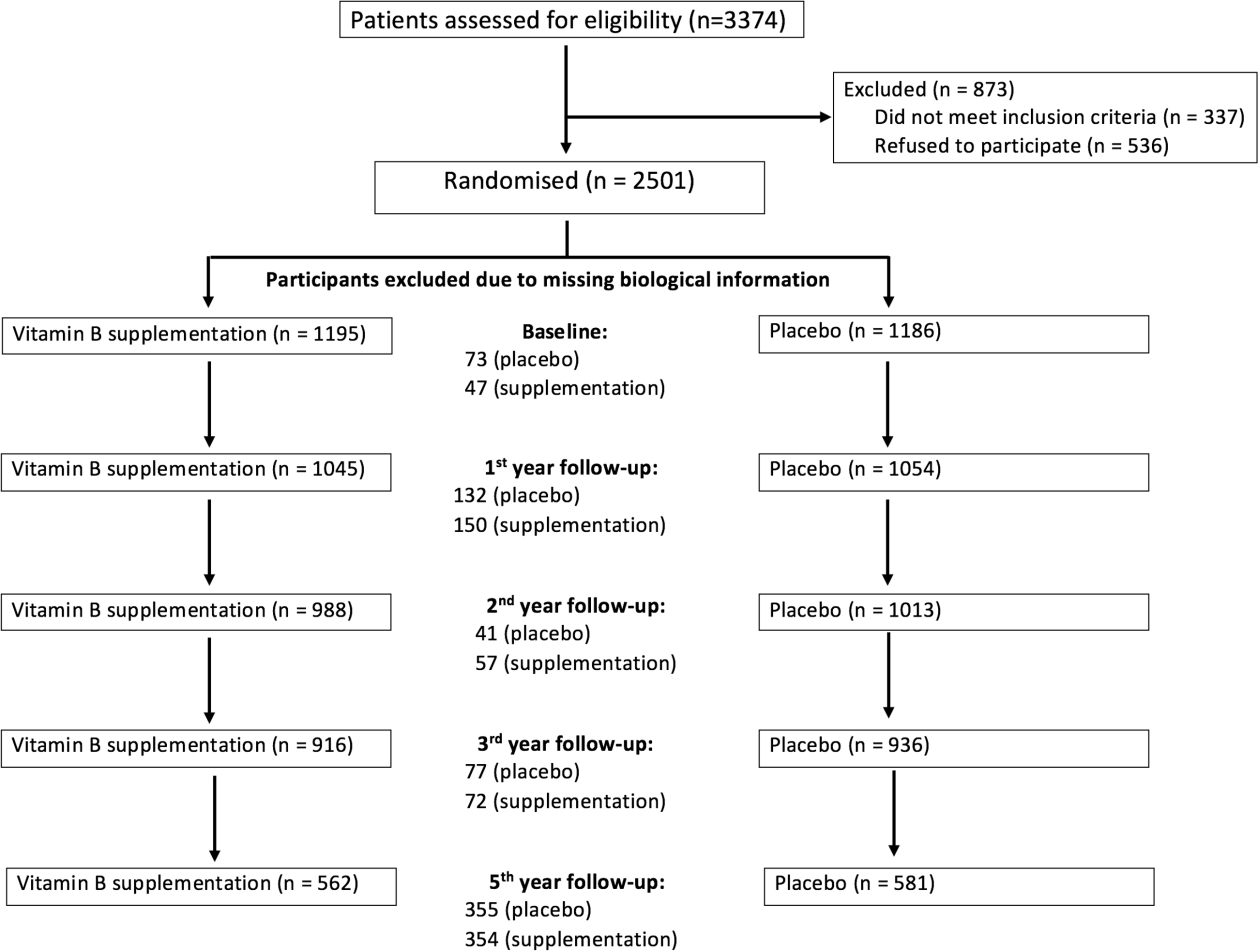
Flowchart describing study participant selection for randomization and causes of exclusion throughout the 5 years.

Treatment compliance was assessed by biannual self-administered questionnaires (or via telephone calls by study physicians). Subjects were considered compliant if they took at least 80% of their allocated treatment. Body Mass Index (BMI in kg/m^2^) was calculated at each annual examination using measurements of height and weight obtained by trained staff following standard protocols. Fasting blood samples were obtained at baseline and at each yearly follow-up examination (non-mandatory visits). All biochemical measurements were centralized. Regarding the clinic visits, the sample sizes at baseline and at years 1, 2, 3 and 5 were as follows: n = 2,381, 2,099, 2,001, 1,852 and 1,143, respectively (Fig 1). The number of participants lost to follow up had no impact on the allele distribution (the percentage of subjects with the *TT* variant at each of the five time points was 15.0%, 15.4%, 15.9%, 15.4% and 14.5%, respectively) or on the supplementation (the percentage of supplemented subjects at each of the five time points was: 50.1%, 49.1%, 48.9%, 49.2% and 49.1%, respectively).

Plasma tHcy, serum folate and serum vitamin B_12_ were obtained by a competitive immunoassay with direct chemiluminescence detection; pyridoxal 5’-phosphate (circulating form of vitamin B_6_) was determined in plasma by high-performance liquid chromatography. Genotyping for the genetic variants encoding *MTHFR* was carried out for the C677T mutation. For that purpose, genomic DNA was isolated from peripheral blood leucocytes using a Genomix kit (Talent-Euromedex, Souffelweyersheim, France) and following the manufacturer's instructions. The polymerase chain reaction for the 677C → T mutation was performed according to the Frosst et al. method [32]; it generated a 198 bp fragment using the forward primer 50-TGA AGG AGA AGG TGT CTG CG and the reverse primer 50-AGG ACG GTG CGG TGA GAG TG. The 677C → T mutation created a HinfI recognition sequence resulting in 175- and 23-bp products for the *677T* allele. The enzyme-restricted bands for the *MTHFR C677T* genotypes were identified by a 10% polyacrylamide gel electrophoresis (PAGE) followed by silver staining. Each experimental batch of DNA was analysed in parallel with two control DNA samples with either *MTHFR 677T* or *MTHFR 677C* alleles, to avoid misinterpretation due to a lack of digestion.

### Statistical analyses

**Fig 2:**
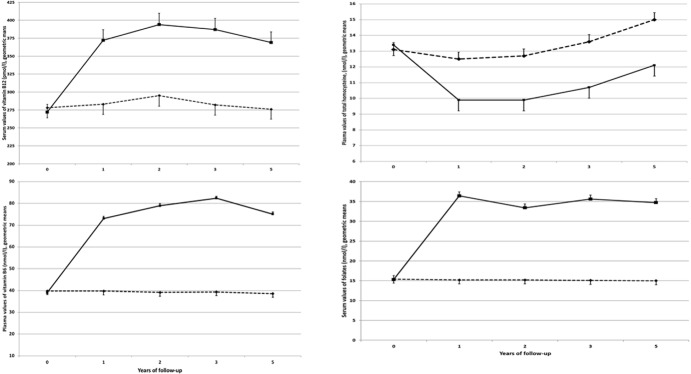
Evolution of the adjusted geometric mean plasma or serum values of vitamin B_6_ (with adjustment for sex, baseline age, plasma creatinine, tHcy and folate), folate (with adjustment for sex, baseline age, MTHFR genotype, tHcy and vitamin B_6_ and vitamin B_12_), vitamin B_12_ (with adjustment for sex, baseline age, tHcy and folate) and tHcy (with adjustment for sex, plasma creatinine, type of prevalent CVD, MTHFR genotype, vitamin B_6_, vitamin B_12_ and folate) from baseline through the 4.7y of follow-up, according to supplementation group. Approximately half of patients not supplemented with B-vitamins and half of patients supplemented with B-vitamins were supplemented with n-3 fatty acids. The groups were merged because the latter supplementation did not interact with B-vitamin supplementation. The SU.FOL.OM3 trial (2003–2009).

**Fig 3:**
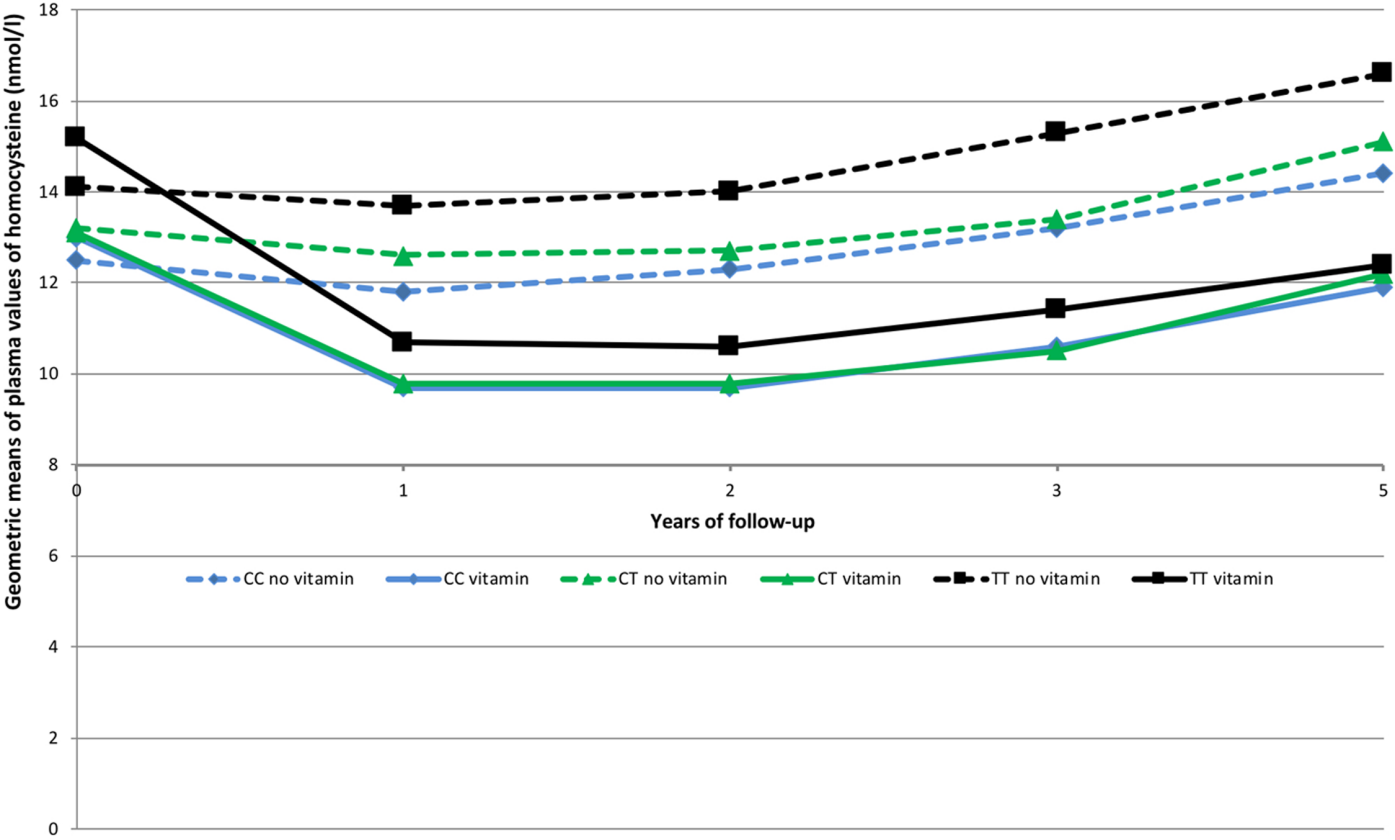
Evolution of the adjusted geometric mean plasma values of homocysteine from baseline through the 4.7 years of follow-up (with adjustment for sex, baseline plasma creatinine, type of prevalent CVD, vitamin B_6_, vitamin B_12_ and folate) among the three MTHFR genotypes and by supplementation group. Approximately half of patients not supplemented with B-vitamins and half of patients supplemented with B-vitamins were supplemented by n-3 fatty acids. The groups were merged because n-3 fatty acids supplementation did not interact with B-vitamin supplementation. The SU.FOL.OM3 trial (2003–2009).

Departure from the Hardy-Weinberg equilibrium was evaluated using a χ^2^ test with one degree of freedom. Hcy, folate, vitamins B_6_ and B_12_ were not normally distributed and were log transformed to approach normality. Their geometric means are displayed in Table 1 and in Figs 2 and 3. Baseline characteristics of participants were compared between men and women and across *MTHFR* C677 genotype using the Student’s *t*-test, analysis of variance, Wilcoxon-Mann-Whitney or Pearson's chi-squared tests, as appropriate.

**Table 1:** Baseline characteristics of participants by gender and MTHFR genotype. The SU.FOL.OM3 trial (2003–2009).

Characteristic	Gender			Genotype			
	Women	Men	p	*TT*	*CT*	*CC*	p
N	482	1899		356	1129	896	
Age (years)	63.1 (9.8)	60.8 (8.8)	0.0001	61.2 (8.8)	61.6 (9.0)	60.9 (9.2)	0.19
BMI (kg/m^2^)	27.5 (5.4)	27.6 (3.6)	0.83	27.8 (4.1)	27.6 (3.9)	27.4 (4.1)	0.26
Total cholesterol (mmol/l)	4.9 (1.0)	4.6 (1.1)	0.0001	4.6 (1.1)	4.7 (1.1)	4.6 (1.0)	0.23
Triglycerides (mmol/l)	1.4 (0.8)	1.5 (0.9)	0.04	1.5 (0.8)	1.5 (0.9)	1.4 (0.8)	0.47
Homocysteine (μmol/l)	13.1 (5.2)	14.3 (6.4)	0.0002	16.3 (9.6)	13.9 (5.1)	13.4 (5.6)	0.0001
Serum folate (ng/ml)	8.0 (3.7)	7.2 (3.5)	0.0001	6.6 (3.7)	7.4 (3.6)	7.6 (3.3)	0.0001
Plasma B_6_ (nmol/l)	43.2 (35.2)	46.2 (36.0)	0.096	46.1 (31.9)	44.8 (33.3)	46.4 (40.2)	0.61
Serum B_12_ (pg/ml)	424 (212)	400 (220)	0.026	400 (210)	401 (245)	404 (184)	0.85
Creatinine (μmol/l)	79.6 (14.6)	82.1 (15.8)	0.0001	78.7 (15.6)	80.2 (17.1)	79.1 (15.7)	0.19
Gender (% male)	—	—		79.3	79.8	80.6	0.86

Except for B_6_ and B_12_ (geometric means), values are means (standard deviation) or percentages.

P values were based on chi-squared, Student’s *t* tests or analysis of variance, as appropriate.

BMI = Body mass index.

Multivariate and linear mixed models (covariance structure: autoregressive) were used to study the baseline relationships among concentrations of tHcy, folate, vitamins B_6_ and B_12_ and genotype and their trend over time. In these models, the outcome variables were the log-transformed plasma or serum tHcy, folate, vitamin B_6_ and vitamin B_12_ at baseline, respectively. The models were mutually adjusted for the remaining variables (i.e., those not modelled as the respective dependent variable), genotype and for baseline variables associated with the outcome of interest. The beta coefficients obtained from these models were further exponentiated. Beta coefficients above one are interpreted as a (β - 1)*100% increase in the outcome when the dependent variable increases by one unit (continuous variable), or compared to the reference category. Beta coefficients below 1 are interpreted as a (1- β)*100% decrease in the outcome when the dependent variable increased by one unit (continuous variable), or compared to the reference category. To compute the p-trends, time in years was considered as a continuous variable among the supplemented and the non-supplemented participants.

Linear mixed models (covariance structure = autoregressive) with repeated measures were used to study whether the effect of supplementation with B-vitamins was different across genotype. The outcome variables were log-transformed values of plasma tHcy, folate, vitamin B_6_ and vitamin B_12_, respectively, at baseline and at years 1, 2, 3 and 5 of follow-up. The models included the baseline value of the respective outcome variable, age, sex, intervention group, time point, and interaction terms for time point and each covariate.

In sensitivity analyses, the same statistical models were fit in the subsample of 1,143 participants with complete data on plasma and serum values of tHcy and B-vitamins over the entire follow-up. All the analyses were performed using SAS software (version 9.4; SAS institute Inc.).

## Results

### Baseline characteristics of the subjects

Compliance with the supplementation regimen was high. In total, 86% of the subjects were considered compliant with the supplementation (reported taking > 80% of the assigned capsules) and compliance was similar (~ 86%) across the four treatment groups. In turn, the overall return rate of completed questionnaires was 99%, 96%, 94% and 95% at 6, 12 and 24 months and at the end of the trial, respectively.

None of the baseline characteristics was statistically different according to supplementation group. Among the 2,381 subjects genotyped, 14.9% were homozygous for the *MTHFR* 677 *TT* variant, 47.4% were heterozygous (*CT*) and 37.7% were *CC* homozygous (Table 1). The observed SNP genotype frequencies conformed to the Hardy-Weinberg proportions (p = 0.57). The mean baseline age was 61.3 y (SD = 9 y). Myocardial infarction was the most prevalent CVD (45.9%), followed by unstable angina (28.2%). The mean tHcy concentration was 13.2 umol/l at baseline. Men were younger than were women (mean age = 60.8 y in men, and 63.1 y in women). Also, men had higher tHcy concentrations and lower folate and vitamin B_12_ concentrations than did women. *TT* homozygous participants had higher plasma tHcy and lower plasma folate concentrations (p<0.0001) than did individuals with the *CC* or *CT* genotype.

### Factors associated with tHcy, folate, vitamins B_6_ and B_12_ at baseline

At baseline, multivariate regression analyses (**Table 2**) showed that plasma tHcy concentration was associated with age (p = 0.001), creatinine concentration (p = 0.001), prevalent CVD (3% lower in subjects with a history of unstable angina (p = 0.03) or 6% lower in subjects with a history of myocardial infarction (p = 0.001) compared with subjects with a history of ischemic stroke), and *MTHFR* genotype (19% higher in subjects with TT genotype compared with those with CC genotype, p = 0.001). It was negatively associated with serum concentrations of folate and vitamins B_6_ and B_12_ (all p < 0.001).

**Table 2:** Factors associated with baseline plasma total homocysteine, vitamin B6, folate and vitamin B12 in multivariate linear regression models, The SU.FOL.OM3 trial (2003–2009).

Outcomes Predictors	Plasma tHcy		Plasma vitamin B_6_		Serum folate		Serum vitamin B_12_	
	β ± SEM	p	β ± SEM	p	β ± SEM	p	β ± SEM	p
**Vitamin supplementation group**		**0.15**		**0.36**		**0.93**		**0.18**
No	Ref		Ref		Ref		Ref	
Yes	1.02 ± 1.01		0.98 ± 1.01		0.999 ± 1.02		0.98 ± 1.01	
**Gender**		**0.27**		**0.009**		**0.001**		**0.005**
Men	Ref		Ref		Ref		Ref	
Women	0.98 ± 1.01		0.94 ± 1.03		1.09 ± 1.02		1.06 ± 1.02	
**Age, years**	1.004 ± 1.000	0.001	0.99 ± 1.00		1.006 ± 1.000	0.001	0.995 ± 1.000	0.001
**Plasma creatinine**	1.005 ± 1.000	0.001	1.003 ± 1.00	0.001	1.000 ± 1.000	0.50	1.001 ± 1.000	0.01
**Recent history of CVD**		**0.002**		**0.62**		**0.25**		**0.04**
Ischemic stroke	Ref		Ref		Ref		Ref	
Unstable angina	0.97 ± 1.01	0.03	1.02 ± 1.02		1.01 ± 1.02		0.95 ± 1.02	0.01
Myocardial infarction	0.94 ± 1.01	0.001	1.02 ± 1.02		1.03 ± 1.02		0.97 ± 1.02	0.10
***MTHFR* genotype**		**0.001**		**0.08**		**0.001**		**0.59**
CC	Ref		Ref		Ref		Ref	
CT	1.02± 1.01	0.10	0.99 ± 1.02		0.968 ± 1.02	0.06	0.99 ± 1.02	
TT	1.19 ± 1.01	0.001	1.06 ± 1.03		0.88 ± 1.02	0.001	1.01 ± 10.2	
**Homocysteine (μmol/l)**	—-		0.99 ± 1.01	0.003	0.98 ± 1.00	0.001	0.99 ± 1.00	0.001
**Serum folate (ng/ml)**	0.98 ± 1.00	0.001	1.03 ± 1.00	0.001	—-		1.01 ± 1.00	0.001
**Plasma B**_**6**_ **(nmol/l)**	0.999 ± 1.000	0.001	—		1.004 ± 1.000	0.001	1.001 ± 1.000	0.07
**Serum B**_**12**_ **(pg/ml)**	0.999 ± 1.000	0.001	1.000 ± 1.09	0.23	1.0002 ± 1.000	0.001	—-	

SEM: Standard error of the mean.

The beta coefficients are interpreted differently if they are above or below the value 1.

Beta coefficients above one are interpreted as a (β -1)*100% increase in the outcome when the dependent variable increased by one unit (continuous variable), or compared to the reference (categorical variable).

Beta coefficients below 1 are interpreted as a (1- β)*100% decrease in the outcome when the dependent variable increased by one unit (continuous variable), or compared to the reference (categorical variable).

Plasma vitamin B_6_ concentrations were 6% higher in men compared with women, were negatively associated with age (p = 0.001) and positively associated with concentrations of creatinine (p = 0.001) and folate (p = 0.001). Serum folate concentrations were 9% higher in women compared with men, positively associated with age (p = 0.001), plasma vitamin B_6_ (p = 0.001) and serum vitamin B_12_ (p = 0.001). TT homozygous individuals had a 12% lower serum folate concentrations compared to CC homozygous individuals. Finally, serum cobalamin concentration was positively associated with creatinine (p = 0.01) and folate (p = 0.001) concentrations and negatively correlated with tHcy, age, type of prevalent CVD and male sex (all p<0.04). At baseline, *MTHFR* genotype was independently associated with plasma tHcy and folate concentrations. *MTHFR* genotype accounted for approximately 4% of the explained variance in folate concentration and 7% in homocysteine concentration.

### Effect of supplementation with B vitamins on tHcy concentration

Owing to the trial’s factorial design, we first tested for interaction between the two types of supplements (n-3 fatty acids and B-vitamins) regarding the evolution of plasma tHcy concentration. As the interaction term was not significant (data not shown), subjects in the two groups receiving B-vitamin supplements (with or without n-3 fatty acids) were pooled. Fig 2 shows the evolution of adjusted mean values of vitamin B_6_, folate, vitamin B_12_ and tHcy by B-vitamin supplementation group (yes/no). In multivariate analyses, the statistical adjustment included variables significantly associated with baseline B-vitamin and tHcy values (Table 2).

At baseline, the values were not significantly different (all p values > 0.27) between the two supplementation groups. All the values were significantly different (p < 0.0001) at 1, 2, 3 and 5 years post-baseline between the two supplementation groups. Among the non-supplemented subjects, concentrations of vitamin B_6_ (p_trend_ = 0.88), folate (p_trend_ = 0.65) and vitamin B_12_ (p_trend_ = 0.55) did not change over time, while concentrations of tHcy (p_trend_<0.001) significantly increased. Among supplemented subjects, plasma values of all vitamins tended to plateau after the first year of supplementation while concentrations of tHcy significantly increased (ptrend<0.001)

Among subjects not supplemented with B vitamins, concentrations of vitamin B_6_ (p_trend_ = 0.88), folate (p_trend_ = 0.65) and vitamin B_12_ (p_trend_ = 0.55) did not vary over time, while concentrations of tHcy (percent change per year = +3%, p_trend_<0.001) significantly increased over time. The trends regarding tHcy concentration were not significantly different by *MTHFR* genotype (tHcy: p for interaction = 0.29, Fig 3). Plasma values of tHcy of TT homozygous subjects were systematically 19% higher compared with those of CC subjects (p<0.001).

At baseline, tHcy was not significantly different between individuals with CC and CT genotype (p = 0.072 for non-supplemented subjects and 0.93 for supplemented subjects), and was higher in individuals with TT compared with CT genotype (p = 0.002 for non-supplemented subjects and 0.001 for supplemented subjects). During follow-up, tHcy concentrations significantly increased among non-supplemented subjects (p_trend_<0.001, with no interaction between time and MTHFR genotype (p for interaction = 0.12). Among supplemented participants, after a more marked decrease in tHcy concentrations in TT subjects (p for interaction < 0.02) over the first year, the same trend was found as that in non-supplemented participants.

Among the subjects supplemented with B-vitamins, concentrations of all studied vitamins increased over the first year (all p values<0.0001), and then tended to plateau (Fig 2). The same trend was found among *MTHFR* categories (data not shown). Also, plasma values of tHcy significantly decreased by 26.3% after the first year of supplementation (p<0.0001, Fig 2), then gradually increased over the subsequent years (linear trend from year 1 to year 5: +5.0% per year, p<0.001). The same trend was found among *MTHFR* categories (Fig 3). However, the reduction in tHcy after the first year of supplementation was higher in TT homozygous subjects than in subjects with the other two genotypes (p_interaction_<0.02). At baseline, the difference in tHcy between TT homozygous and CC homozygous subjects was = 2.33 μmol/l (p<0.001). At the end of the supplementation period, that difference was reduced to 1.06 μmol/l while remaining statistically significant (p<0.001).

Results of the sensitivity analyses (n = 1,143 subjects with complete biomarker data) were consistent with those obtained in the main analysis.

## Discussion

To our knowledge, this is the first study reporting findings on effect modification by *MTHFR* genotype of the association between a long-term B-vitamin supplementation (560 μg of 5-methyl-THF, 3 mg vitamin B_6_ and 20 μg of vitamin B_12_) and plasma values of tHcy in adults with CVD history. We found that baseline concentrations of folate and tHcy were associated with different *MTHFR* 677 genotypes. Throughout the supplementation, the *MTHFR* 677 TT genotype was associated with lower serum folate concentrations and higher plasma tHcy concentrations than were the CT or CC genotype. *MTHFR* 677 genotype was an independent predictor of response to supplementation, and it interacted with it by lowering plasma tHcy values after year 1. This study, therefore, could help inform the debate regarding 5-MTHF fortification for public health reasons and regarding folic acid supplementation for the prevention of neural tube defects or congenital CVD[33–36]. Another implication of our results concerns migraine treatment. Menon et al. [14, 15] have demonstrated that supplementation with B vitamins to relieve headaches caused by migraine was more efficient in carriers of the C677T variant. Thus, MTHFR genotype may be a consideration if B-vitamin supplementation is to be used as a migraine treatment. Our data also support suggestions from other observational and experimental studies [25, 26, 37, 38] that TT homozygotic individuals have greater folate requirements than do their CC or CT counterparts. However, active B-vitamin supplementation produces tHcy concentrations in TT homozygous individuals that are closer to the concentrations found in CC homozygotous individuals.

An interesting observation of the present study was the fact that folate status improved moderately even in the non-supplemented group, perhaps due to dietary changes in this cohort of patients. As we did not assess dietary intake of B-vitamins over time, this hypothesis could not be confirmed. However, there are no reasons to suspect that this change (if indeed present) could differ according to the B-vitamin supplementation group. We collected information on dietary supplement use (including folate) outside of that provided by the supplementation under study. Such use was not significantly different between the placebo and the supplemented group, and could also partly explain the above-mentioned folate status improvement. Nevertheless, a more notable impact on folate levels was seen after B vitamin supplementation, such that mean serum folate levels more than doubled after the first year of supplementation. As expected, tHcy decreased as folate levels increased.

We found that subjects homozygous for the 677T allele had higher tHcy concentrations compared with the CC/CT genotype before and after B vitamin supplementation, even though the magnitude of the difference significantly decreased over the course of the supplementation. At the end of the 4.7 years of follow-up, supplemented subjects still had lower tHcy concentrations than did non-supplemented ones, and also had lower tHcy concentration compared with those measured at the start of the study (Fig 2). Indeed, many investigators have reported lower folate concentrations in TT subjects than in CC or CT subjects [29, 39, 40]. These results were confirmed by two meta-analyses of intervention studies performed recently [41, 42]. Colson et al. demonstrated that a short-term supplementation with folate affected plasma homocysteine and serum folate differently according to MTHFR status [41]. Tsang et al. studied healthy women aged 12–49 y and reported that low blood folate concentrations were associated with the MTHFR C677T mutation [42]. As B vitamin supplementation reduced the difference in tHcy concentration between TT and CT (or CC) subjects (Fig 2), our results support the fact that B vitamins provided as low-dose supplements may help in lowering tHcy concentrations in subjects with 2 T alleles. A major objective of the present study was to examine how the effect of B vitamin supplementation on tHcy concentrations was moderated by the *MTHFR* 677C→T mutation. After the first year of supplementation, TT homozygous participants experienced a significantly lower absolute decrease in tHcy concentrations than did CC or CT participants. In fact, the tHcy concentration was lower in TT homozygous participants supplemented with B-vitamins than in CC or CT participants not supplemented with these vitamins (Fig 2). This gene-nutrient interaction has been described by some authors[25, 29, 37, 38, 43, 44]. Tsaï et al. [38] investigated the effect of the *MTHFR* 677 C →T variant on tHcy concentrations before and after fortification of grain products with folic acid in a sample of 884 Caucasian and 587 African American subjects. They found a gene-nutrient interaction and recommended the measurement of tHcy concentration rather than genotyping *MTHFR* 677 TT as the primary assay for diagnosis and monitoring of moderate hyperhomocysteinemia. Forh et al. [37] conducted a randomized controlled trial during which 160 women were supplemented with folate over 8 weeks. The authors found that women with the TT genotype exhibited a greater decrease in tHcy concentrations following vitamin supplementation. For the studies without gene-nutrient interaction findings, lower values of folate and higher baseline values of tHcy concentrations were given as explanations. Selhub et al. have suggested that the tHcy-lowering effect of folate reaches a plateau at intakes of around 400 ug/day, with higher doses of folate producing little additional benefit [45].

Important strengths of this study include its design, the large sample size, long treatment duration, and the low-dose supplementation with three different B-vitamins known to impact reduction of tHcy concentration. Most of the studies in the literature have used either the three B-vitamins separately [25, 29, 37, 38, 43, 44], or a combination of two among the three vitamins [46, 47]. In fact, plasma B-vitamins are not the preferred markers of B-vitamin status. Next, this study used 5-methy-THF, which can be put into supplements, but not used in fortification, because it is not heat-stable. The daily dosage of 5-methyl-THF used as vitamin supplementation is about 3 times higher than the usual daily dosage of folic acid obtained from mandatory fortification (mean < 150 micrograms/day) in the United States [48]. Therefore, the same magnitude of effect cannot be expected. Different assay methods for measuring folate provide somewhat different results[49–51]. Therefore, caution should be used when comparing absolute values obtained in our population with other populations. However, as the same assay method was used all along the trial, it did not influence the time trends of folate values. Also, as a dynamic measure reflecting recent nutritional intake, plasma B-vitamin values fluctuate; thus, they are normally used as a marker of short-term B-vitamin status. The participants in this study had a personal history of CVD. This may impede the extrapolation of our results to a general population. However, the magnitude of this bias is limited by the fact that prevalent CVD does not have an impact on plasma values of either tHcy or B-vitamins. Red blood cell folate and other vitamins are regarded as markers of long-term B-vitamin status because they reflect B-vitamin status during the erythropoiesis. Shorter and more frequent blood sampling intervals, especially early in the intervention, would have provided more data on the timing and the trajectories of genotype-dependent responses to B-vitamin supplementation. There was a reduction over time of the number of participants for whom blood samples were available. This led to a reduction of the statistical power when comparing the prevalence of hyperhomocysteinemia at the end of the study.

In summary, this study confirmed that reduction in plasma tHcy concentration by B-vitamin supplementation could be influenced by *MTHFR* genotype. Subjects with the TT genotype may be able to compensate for the effects of the *677C→T* mutation on tHcy and folate metabolism if their B-vitamin status is adequate. These aspects must be considered in food fortification and supplementation policy development.

## Supporting information

S1 File CONSORT-2010-checklist. (DOC)

S2 File SUFOLOM3 protocol in English. (DOC)
