# Supplementary material for: MTHFR 677C → T genotype modulates the effect of a 5-year supplementation with B-vitamins on homocysteine concentration: The SU.FOL.OM3 randomized controlled trial
Source: PLoS One. 2018 May 29;13(5):e0193352. doi: 10.1371/journal.pone.0193352 (PMC5973566; doi:10.1371/journal.pone.0193352)
Supplement: S2 File — (DOC) [file pone.0193352.s002.doc]

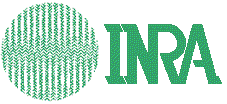

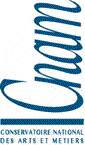


| **Unité 557 Inserm/Unité 1125 Inra/CNAM**  Nutrition et maladies chroniques: épidémiologie  et intervention en Santé Publique |  | Institut Scientifique et Technique de la Nutrition et de l’Alimentation(Istna)  **UMR Inserm/Inra/Cnam** |
| --- | --- | --- |

**RESEARCH PROTOCOL**

Prevention study “ SU.FOL.OM3*”*

*sECONDARY PREVENTION TRIAL DEVELOPED TO TEST THE impact OF supplEmentation WITH FOLIC ACID vitamins B6 AND B12 AND/OR OMEGA 3 FATTY ACIDS ON THE PREVENTION OF RECURRENT ISCHEAMIC EVENTS IN SUBJECTS WITH ATHEROSCLEROSIS IN THE coronarY OR CEREBRAL ARTERIES*

***This study is supported by***

The French cardiology society (la Société Française de Cardiologie)

The French federation of cardiology (la Fédération Française de Cardiologie)

The national college of cardiology (le Collège National des Cardiologues)

***This study is coordinated by:***

***UMR U 557 Inserm/ U 1125 Inra / CNAM***

Pilar Galan (coordinator), Louise Mennen, Geneviève Potier de Courcy,

Paul Preziosi, Sandrine Bertrais, Angelika de Bree, Serge Hercberg (director)

***This study is promoted by*:**

**INSERM**

Institut Scientifique et Technique de la Nutrition et de l'Alimentation, ISTNA,

ConservatoireNational des Arts et Métiers (CNAM), 5 rue Vertbois, F-75003 Paris

Tel: 01-53-01-80-41 ; Fax 01-53-01-80-70

s_galan@vcnam.cnam.fr ; hercberg@vcnam.cnam.fr

**Coordinating committee of SU.FOL.OM3**

**UMR U 557Inserm/ U 1125 Inra / CNAM**

- Pilar Galan, research director of INRA (principal investigator)
- Geneviève Potier de Courcy, representative of CNRS
- Louise Mennen, researcher of ISTNA/Cnam
- Paul Preziosi, researcher of ISTNA/Cnam
- Sandrine Bertrais, researcher of ISTNA/Cnam
- Angelika de Bree, researcher of ISTNA/Cnam
- Serge Hercberg, research director of INSERM (director of UMR Inserm/Inra/Cnam)

**Strategic committee of SU.FOL.OM3**

- Pierre Amarenco (Hôpital Lariboisière, Paris)
- Jacques Blacher (Hôpital Broussais, Paris)
- Serge Briançon (CHU Nancy)
- Mireille Brochier (CHU Tours)
- Eric Bruckert (CHU Pitié-Salpètrière, Paris)
- Nicolas Danchin (Hôpital Georges Pompidou, Paris)
- Pierre Ducimetière (Inserm, U 258, Villejuif)
- Véronique Ducros (CHU Grenoble)
- Alain Favier (CHU Grenoble)
- Alain Grynberg (INRA, Paris)
- Jean-Louis Guéant (CHU Nancy)
- Jean-Claude Guilland (CHU Dijon)
- Louis Guize (Hôpital Georges Pompidou, Paris)
- Michel Krempf (CHU Nantes)
- Gérard Jullien (Collège National des Cardiologues, Marseille)
- Denis Lairon (Inserm, U 476, Marseille)
- Gisèle Le Moël (Hôpital Bichat, Paris)
- Alain Lemoine (Hôpital Nevers)
- Jean-René Lusson (CHU Clermont-Ferrand)
- Nicole Moatti (Hôpital Georges Pompidou, Paris)
- François Paillard (CHU Rennes)
- Isabelle Queré (CHU Montpellier)
- Alberto Rhigetti (Hôpital cantonal de Genève)
- Monique Romont-Rousseau (CHU Lille)
- Marie-Pierre Sauvant (CRNH Clermont-Ferrand)
- Jean-Louis Schlienger (CHU Strasbourg)
- Chantal Simon (CHU Strasbourg)
- Daniel Thomas, (CHU Pitié-Salpètrière, Paris)

**Contents**

1. Introduction [4](#__RefHeading___Toc2418997)

1.1. The public health impact of cardiovascular diseases [4](#__RefHeading___Toc2418998)

1.2. B-vitamins, homocysteine and cardiovascular diseases [4](#__RefHeading___Toc2418999)

1.2.1. Homocysteine [4](#__RefHeading___Toc2419000)

1.2.2. Homocysteine and vascular disease [4](#__RefHeading___Toc2419001)

1.2.3. Mechanism by which homocysteine increases the risk of vascular disease [5](#__RefHeading___Toc2419002)

1.2.4. Determinants of homocysteine [6](#__RefHeading___Toc2419003)

1.2.5. Intervention trials [6](#__RefHeading___Toc2419004)

1.3. The relation between n-3 fatty acids and cardiovascular diseases [7](#__RefHeading___Toc2419005)

1.3.1. Omega-3 fatty acids [8](#__RefHeading___Toc2419006)

1.3.2. Omega-3 fatty acids and vascular disease [8](#__RefHeading___Toc2419007)

1.3.3. Mechanism by which omega-3 fatty acids decrease the risk of vascular disease [8](#__RefHeading___Toc2419008)

1.3.4. Intervention trials [9](#__RefHeading___Toc2419009)

1.4. Conclusion and implications for future research [9](#__RefHeading___Toc2419010)

2. Objectives of su.fol.om3 [10](#__RefHeading___Toc2419011)

3. Design of su.fol.om3 [10](#__RefHeading___Toc2419012)

4. Participants [11](#__RefHeading___Toc2419013)

4.1. Sampling frame [11](#__RefHeading___Toc2419014)

4.2. Number of participants [11](#__RefHeading___Toc2419015)

4.3. Eligibility criteria [12](#__RefHeading___Toc2419016)

4.3.1.Inclusion criteria [12](#__RefHeading___Toc2419017)

4.3.2. Exclusion criteria [12](#__RefHeading___Toc2419018)

4.4. Recruitment of patients [12](#__RefHeading___Toc2419019)

4.4.1. Eligibility judged by cardiologists and neurologists [12](#__RefHeading___Toc2419020)

4.4.2. Responsibility of the cardiologists and neurologists [12](#__RefHeading___Toc2419021)

4.4.3. Screening questionnaire [13](#__RefHeading___Toc2419022)

4.4.4. Recruitement period [13](#__RefHeading___Toc2419023)

5. Registration and randomization by su.fol.om3 team [13](#__RefHeading___Toc2419024)

5.1. Registration [13](#__RefHeading___Toc2419025)

5.2. Randomization [13](#__RefHeading___Toc2419026)

6. Treatment [14](#__RefHeading___Toc2419027)

6.1. Treatment regimen and compliance [14](#__RefHeading___Toc2419028)

6.2. Length of treatment [14](#__RefHeading___Toc2419029)

6.3. Supplements and packaging [14](#__RefHeading___Toc2419030)

7. Monitoring of the participants [14](#__RefHeading___Toc2419031)

7.1. Annual check-up [14](#__RefHeading___Toc2419032)

7.2. Follow-up questionnaire for participants [15](#__RefHeading___Toc2419033)

7.3. Follow-up by clinicians [15](#__RefHeading___Toc2419034)

7.4. Biological parameters to be measured [15](#__RefHeading___Toc2419035)

7.5. Measurement of intermediate endpoints [16](#__RefHeading___Toc2419036)

7.6. Hard endpoints [16](#__RefHeading___Toc2419037)

7.7. Validation of endpoints [16](#__RefHeading___Toc2419038)

8. Statistical analyses [17](#__RefHeading___Toc2419039)

9. Sources of bias and issues that may affect the power of the study [17](#__RefHeading___Toc2419040)

9.1. Unexpected unblinding of the protocol [17](#__RefHeading___Toc2419041)

9.2. Compliance problems [17](#__RefHeading___Toc2419042)

9.3. Loss to follow-up [17](#__RefHeading___Toc2419043)

10. Mobilization and motivation of participating health professionals [18](#__RefHeading___Toc2419044)

10.1. Mobilization of clinicians [18](#__RefHeading___Toc2419045)

10.2. Motivation of clinicians and other health professionals [18](#__RefHeading___Toc2419046)

11. Time schedule [19](#__RefHeading___Toc2419047)

12. References [19](#__RefHeading___Toc2419048)

# 1. Introduction

## 1.1. The public health impact of cardiovascular diseases

Currently cardiovascular diseases are the major cause of mortality in France. Each year ~32% (or 170 000 subjects) of all deaths in France are due to cardiovascular diseases (HCSP, 2000). Of all cardiovascular diseases, coronary heart disease is the most important; it is responsible for 27% of all cardiovascular disease deaths (45 000 subjects). Another important category of vascular diseases are the cerebrovascular accidents (25%, 42 000 subjects). In France the incidence of (non-)fatal myocardial infarctions and cerebrovascular accidents is estimated at 115 000 (Guize and Richard, 1990), and 130 000 (Roquebrune, 1994) respectively, per year.

Numerous epidemiological studies have identified nutritional factors that may reduce the incidence of cardiovascular diseases (HCSP, 2000). Folate (natural form of folic acid), vitamin B6 and B12 on the one hand and n-3 fatty acids on the other hand are nutritional factors for which there is scientific evidence that they might have a protective effect on the development of cardiovascular diseases.

## 1.2. B-vitamins, homocysteine and cardiovascular diseases

The last couple of years there has been an increasing interest into B vitamins (folate, B6 and B12) because of their role in the homocysteine metabolism. Elevated plasma homocysteine concentrations have been associated with an increased risk of cardiovascular diseases in numerous studies in different study populations and countries (Boushey et al, 1995, Refsum et al, 1998, Ueland et al, 2000).

### 1.2.1. Homocysteine

Homocysteine is a sulfur-containing amino acid that is not used for the synthesis of proteins. Foods only contain traces of homocysteine. Homocysteine is formed when cells metabolize the essential amino acid methionine. Homocysteine was discovered in 1933 by Du Vigneaud (Du Vigneaud, 1933).

### 1.2.2. Homocysteine and vascular disease

In 1969 McCully formulated the hypothesis that the accumulation of homocysteine might lead to arteriosclerosis (McCully, 1969). His hypothesis was based on clinical observations in two children with homocystinuria which derived from different inborn errors but led to similar arteriosclerotic damage. From this publication onwards many epidemiological studies (Boushey et al, 1995; Nygard et al, 1999; Hankey and Eikelboom, 1999) were performed and generally indicated that the homocysteine level in patients suffering from cardiovascular diseases was higher as compared to controls.

Most of the initial investigations had a case-control or retrospective design (Kang et al, 1986; Malinow, 1990; Genest et al, 1990; Ubbink et al, 1991; Pancharuniti et al, 1994; Wu et al, 1994; Dalery et al, 1995; Malinow et al, 1996; Graham et al, 1997). These kind of designs can, however, not exclude that increased homocysteine levels are the result rather than the cause of the disease, because blood samples for these studies are typically taken after the diagnosis of the cardiovascular event. Prospective studies provide stronger evidence for a causal role of elevated homocysteine levels in the etiology of cardiovascular diseases, as collection of blood takes place before the vascular event. The initial associations as found in case-control studies were confirmed in prospective studies (Stampfer et al, 1992; Arnesen et al, 1995; Stehouwer et al, 1998; Nygard et al, 1997; Giles et al, 1998; Wald et al, 1998; Bots et al, 1999; Kark et al, 1999). From different meta-analyses it was estimated that the odds ratio for coronary heart disease for each 5 µmol/l increase in homocysteine varies between 1,2 to 1,9 (Boushey et al, 1995; Danesh and Lewington, 1998; Ueland et al, 2000). Thus, a difference in homocysteine level of 5 µmol/l is associated with a 20 to 90 % difference in risk of coronary heart disease.

### 1.2.3. Mechanism by which homocysteine increases the risk of vascular disease

To date, no definitive mechanism by which homocysteine increases the risk of vascular disease has been identified. However, mostly *in vitro* experiments with very high homocysteine concentrations have identified the stimulation of atherosclerosis and thrombosis through adverse effects on coagulation pathways, platelets, endothelial cells, and vascular smooth muscle cells (Thambyrajah and Townend, 2000)

Despite these critical remarks, to date one of the most plausible mechanisms by which homocysteine may cause atherosclerosis and thrombosis may be by inducing endothelial dysfunction (Thambyrajah and Townend, 2000; McDowell and Lang, 2000; Brown and Hu, 2001). Endothelial cells play a vital role in regulating and maintaining vascular health. In addition, endothelial cells are essential to hemostatic processes of cell adhesion and migration, coagulation and fibrinolysis (Brown and Hu, 2001). A key regulatory system of endothelial cells involves nitric oxide synthase, which synthesizes nitric oxide (NO) (McDowell and Lang, 2000). Endothelial derived NO regulates vessel tone, inhibits platelet activation, adhesion and aggregation, limits smooth muscle cell proliferation and modulates endothelial-leukocyte interaction (Thambyrajah and Townend, 2000).

### 1.2.4. Determinants of homocysteine

Age, sex and genetic factors are non-modifiable determinants of the homocysteine concentration. Especially genetic mutations in genes that encode for key enzymes in homocysteine metabolism can have a large impact on the homocysteine concentration. However, the effect of a common polymorphism in the gene encoding for methylene-tetrahydrofolate reductase (MTHFR) , i.e. the 677 C<T polymorphism (Frosst et al, 1995), can be modified by the folate status; subjects with the homozygous mutant form of this polymorphism (TT) only have an elevated homocysteine level in case of a sub-optimal folate status.

The B-vitamin status is the most important modifiable homocysteine-determinant. Deficiencies of folate, vitamin B6 and B12 can lead to extremely elevated homocysteine concentrations (Ueland and Refsum, 1989). In addition, in the non-deficient range inverse relations have been shown between intakes of folate, vitamin B6 and B12 and homocysteine concentrations (Selhub et al, 1993; de Bree et al, 2001).

Folate is the substrate donor in the remethylation of homocysteine to methionine (Finkelstein, 1990). In contrast, the other B-vitamins are cofactors of enzymes and are not used up during homocysteine degradation; thus, they are not often a limiting factor. Due to its role as substrate donor, folate shows in general the strongest inverse relation with plasma homocysteine levels (de Bree et al, 2001). Additional dietary folate (Brouwer et al, 1999: Riddell et al 2000) and supplementation with folic acid (synthetic form of folate) significantly reduces the homocysteine level of subjects with a normal baseline B vitamin status (Brattstrom et al, 1988; Ubbink et al, 1993; Landgren et al, 1995; Naurath et al, 1995; Rasmussen et al, 1996; Santhosh-Kumar et al, 1997; Woodside et al, 1998; Dierkes et al, 1998; Den Heijer et al, 1998; Bjorkegren and Svardsudd, 1999; Vermeulen et al, 2000a and 2000b).

### 1.2.5. Intervention trials

Because of the homocysteine-lowering effect of folic acid, several studies investigated the effect of folic acid supplementation on intermediate endpoints of vascular damage. Endothelial dysfunction is such an intermediate endpoint. Brown and Hu recently reviewed trials that considered the effect of folic acid supplementation on endothelial function (Brown and Hu, 2001). The general picture that emerges from these studies is that folic acid (5 to 10 mg/d) improves or restores endothelium-dependent vasodilatation and may decrease the chance of thrombosis by reducing levels of coagulation factors in healthy subjects and in patients with high homocysteine levels (Brown and Hu, 2001). The observed benefit is probably largely explained by the lowering of homocysteine concentrations. However, folic acid may also have a beneficial effect on endothelial function independent of the homocysteine concentration (Verhaar et al, 1998; Stroes et al, 2000).

Currently there are 3 non-controlled trials of which 2 show that supplementation with folic acid (5000 µg) and vitamin B6 (250 mg) reduces the risk of cardiovascular events (coronary, peripheral and cerebral) in patients with high homocysteine levels and existing cardiovascular diseases, to the level of patients with existing cardiovascular diseases, but with normal homocysteine levels (De Jong et al, 1999; Vermeulen et al, 2000a). The third trial investigated the effect of folic acid (2500 µg), vitamin B6 (25 mg) and vitamin B12 (250 µg) on the regression of carotid plagues. Vitamin supplementation resulted in a decreased rate of progression of the plague growth in 101 patients with vascular disease with normal and elevated homocysteine levels (Hackam et al, 2000).

Up to date the results of two randomized, placebo-controlled trials are available (Vermeulen et al, 2000b; Schnyder et al, 2001). Both trials used intermediate endpoints. Vermeulen et al observed fewer abnormal exercise electrocardiography tests after 2 years of supplementation with 5000 µg folate and 250 mg vitamin B6 (n=78) compared to the placebo group (n=80). However, this study was not blinded and the internal validity of the exercise electrocardiography tests is questioned (Bostom and Garber, 2001). Moreover, an effect of treatment on other surrogate outcome measures (ankle-brachial pressure index and duplex-scanning of the carotis and peripheral arteries) was not observed (Vermeulen et al, 2000a). The intervention study of Schnyder et al was double-blind and investigated the effect of a daily combination (1 mg) of folic acid, vitamin B12 (µg 400) and pyridoxine (10 mg) on the rate of the restenosis as assessed by quantitative coronary angiography. After 6 months of supplementation, the intervention group showed a significant reduction of the rate of restenosis (Schnyder et al, 2001)

## 1.3. The relation between n-3 fatty acids and cardiovascular diseases

During the last 30 years an increasing interest has been raised into the essential n-3 fatty acids, as several studies suggest that they play a major role in the prevention and modulation of certain chronic diseases, like cardiovascular diseases. The effects of n-3 fatty acids against coronary damage are well documented by experimental studies with cellular and tissue models, and in animal and human intervention studies (Connor, 2000).

### 1.3.1. Omega-3 fatty acids

Omega-3 fatty acids are natural components in animal and plant foods. They exist of a carbon chain that on one end contains a methyl group and on the other hand a functional acid. These fatty acids are called polyunsaturated (PUFA) as they contain more than one double bindings. The n-3 PUFAs have there first double binding situated at the third carbon atom calculated from the methyl group end (Passmore and Eastwood, 1986)

The omega-3 derive either from plant oils (oil of colerape, soja and nuts), or from animal oil (fish and fish oil). Fatty fish (mackerel, haring, salmon) is a good source of long chain omega-3 fatty acids (Passmore and Eastwood, 1986).

### 1.3.2. Omega-3 fatty acids and vascular disease

The first hypothesis considering n-3 fatty acids derives from observations among Eskimos from Greenland, as the low incidence of coronary heart disease in this population was ascribed to the large consumption of fatty fish, which is an important source of omega-3 long chain fatty acids (Dyerberg and Bang, 1982). Consumers of this type of fish have elevated plasma levels of docosahexaenoic acid (DHA) and eicosapetaenoic acid (EPA) and lower levels of arachidonic acid (Bang et al, 1980).

Several epidemiological studies have observed an inverse relation between regular fish consumption and the incidence of cardiovascular diseases (Kromhout, 1985; Siscovick et al, 1995; Vollset et al, 1985, Albert et al, 1998; Ascherio et al, 1995; Oomen et al, 2000). From one of these prospective studies it was estimated that one serving of fish per week reduced the risk of total and cardiovascular mortality with respectively 30 and 45 % Furthermore, one serving a week of fatty fish reduced the risk of cardiac arrests with 50% (Albert et al, 1998).

### 1.3.3. Mechanism by which omega-3 fatty acids decrease the risk of vascular disease

Omega-3 fatty acids are thought to prevent cardiovascular diseases by their role in platelet aggregation (via synthesis of eicosanoides), resulting in an antithrombotic effect (Gester, 1995). Furthermore, there are studies that indicate that n-3 fatty acids may have an anti-arrythmic effect (Segal-Isaacson and Wylie-Rosett, 1999). Finally, omega-3 fatty acids have a blood pressure lowering effect in subjects with hypertension; probably trough an effect on the fluidity of the endothelial membrane, which is important for constriction and dilatation of the arterial inner layer (Knapp, 1989; Knapp, 1990; Morris et al, 1992; Appel et al, 1993; Gerster et al, 1993).

More specifically, EPA and DHA intervene in hemostase and vasomotoric processes by affecting the expression of enzymes that synthesize eicosanoides (Kramer et al, 1996; Lemaitre et al, 1997). Furthermore, EPA may have properties to intervene in inflammatory processes, blood flow regulation, ion transport, and modulation of synaptic transmission (Kang et al, 1996; Grynberg et al, 1997; Nair et al, 1997). The long chain n-3 fatty acids intervene by reducing the triglyceride synthesis in the liver and by decreasing secretion of triglycerides rich VLDL (Nenseter et al, 1992; Gotlicher et al, 1992; Keller et al, 1993; Bordin et al, 1998; Haglund et al, 1998; Lu et al, 1999).

### 1.3.4. Intervention trials

Intervention trials support the results of observational studies. The Lyon study (De Lorgeril et al, 1999) indicated that a Mediterranean diet, which is rich in omega-3 fatty acids, results in a reduction of all coronary events of at least 66 % and in a reduction of total mortality of 76 %. DART (Diet and Reinfarction Trial), showed a smaller risk reduction of 29 % in men who consumed at least 300 g fish per week compared to men who do not eat fish at all. However, no statistically significant protective effect of fish consumption was observed for primary cardiovascular events (Burr et al, 1989). The results of these dietary intervention trials are supported by the results of a non-placebo controlled, non-blinded intervention trial with omega-3 supplements (Gissi-Prevenzione Investigators, 1999). This study included patients with a recent myocardial infarction (n=11,324). After 3,5 years of intervention they observed that treatment with n-3 PUFA, significantly lowered the risk of death, non-fatal myocardial infarction, and stroke with 10 to 15%.

## 1.4. Conclusion and implications for future research

Definitive proof that supplementation with B-vitamins or omega-3 fatty acids will lead to a reduced cardiovascular diseases morbidity and/or mortality is still scarce. The currently available intervention trials with mostly intermediary end-points, did either not have a study design that allows this conclusion (De Jong et al, 1999; Gissi-Prevenzione Investigators, 1999; Vermeulen et al, 2000a; Vermeulen et al, 2000b; Hackam et al, 2000) or the results need to be reproduced before they can be regarded as definitive (Schnyder et al, 2001). Furthermore, there is a need for trials with hard end-points like myocardial infarction or cerebrovascular accidents.

Secondary intervention trials with B-vitamins have recently started (Clarke and Armitage, 2000), but not all of these trials used a combination B vitamins and most trials used pharmacological doses. This has the disadvantage that the results are difficult to extrapolate to the general population. In addition, recent research has indicated that supplementation with 5-methyl tetrahydrofolate (5-methyl THF), the most abundant natural folate vitamer, is safe and lowers homocysteine levels (Fohr et al, 2002). This form of folate, in contrast to folic acid, does not lead to circulating unmetabolized folic acid (Kelly et al, 1997). Unmetabolized folic acid is hypothesized to mask the hematological manifestations of a vitamin B12 deficiency, thereby predisposing subjects to irreversible neurological damage. This information on 5-methyl THF was not available when other intervention studies started and therefore they all use folic acid (Clarke and Armitage, 2000).

Taken all this information together, there is a need for a large double-blind placebo controlled randomized intervention trial evaluating the effect of supplementation with B-vitamins (exchanging folic acid for 5-methyl-THF) and n-3 fatty acids in nutritional doses on hard cardiovascular endpoints. Therefore, we propose the following intervention study in which participants are Supplemented with natural Folate, vitamin B6 and B12 and/or Omega-3 fatty acids: the SU.FOL.OM3 study.

# 2. Objectives of su.fol.om3

- Primary objective:

-To test the hypothesis that supplementation with 5-methyl THF (or folic acid), vitamin B6 and B12 and/or omega-3 fatty acids prevents recurrence of ischemic events in patients who already have experienced a coronary or cerebrovascular event.

- Secondary objectives:

-To evaluate the role of certain genetic mutations in the susceptibility of patients to the effects of supplementation;

-To study the impact of supplementation on markers of hemostase, endothelial function and the incidence of arterial and venous thrombosis.

# 3. Design of su.fol.om3

The SU.FOL.OM3 study is a double-blind randomized placebo-controlled secondary intervention trial in which participants are supplemented daily for a period 5 years with nutritional doses of 5-methyl THF (or folic acid) (500 µg), vitamin B6 (3 mg) and B12 (20µg) and/or omega-3 supplements (600 mg with an EPA:DHA ratio of 2:1).

A factorial design will be applied to investigate the separate effects of the B-vitamins, and the omega-3 fatty acids as compared to the placebo. Participants will be randomly allocated to the following treatment groups:

1. 5-methyl THF (or folic acid) vitamin B6 and B12 and a placebo (S1P);
2. omega-3 fatty acids and a placebo (S2P);
3. 5-methyl THF (or folic acid) vitamin B6 and B12 and omega-3 fatty acids (S1S2);
4. two placebos (PP)

# 4. Participants

## 4.1. Sampling frame

The participants are patients with a history of cardiovascular diseases which will be recruited from all regions in France.

## 4.2. Number of participants

Our goal is to be able to observe relatively weak effects with an alpha of 5 % and a power of 90%, testing a one-sided effect. A priori we do not expect an interaction between B-vitamins and n-3 fatty acids, therefore, we will not take this into account while calculating the power of this study.

*Risk reduction in each separate group.* Our hypothesis is to observe a risk reduction of recurrent coronary and cerebrovascular events due to each intervention of 10%, which means a risk of 0.9 in group S1P and S2P and a risk of 0.8 in group S1S2 as compared to the risk of the PP group.

*Baseline risks in each separate group.* The baseline risk of the PP group is 0.087, as estimated by cardiologists. The baseline risk of the S1P and S2P group is thus 0.078 (0.087 x 0.9).

*Risk reduction in combined groups.* To estimate the effect of S1 we will compare the combined group S1P and S1S2 with the combined group PP and S2P. The risk reduction in group S1P and S1S2 is thus estimated at 15% ([10% in group S1P and 20% in group S1S2]/2).

*Baseline risk in combined groups.* The baseline risk in group PP and S2P is: 0.0826 (= [0.078 + 0.087]/2). To estimate the effect of S2 we will compare group S2P and S1S2 with group PP and S1P the baseline risk in group PP and S1P is also: 0.0826 (= [0.078 + 0.087]/2).

For the calculation of the necessary number of patients we have taken into account the comparison of exponential survival distributions (Hill, 1996), an inclusion period of one year and a follow up of 5 years. With these parameters we calculated that a number of 1188 subjects are necessary in each combined group, for a total of 2376 patients .

To make up for drop-outs and patients that are lost to follow-up (10%) the total number of patients that will be included is set at 3000. This number of subjects enables us to identify causal relations with an acceptable statistical power within a protocol that is practically feasible.

## 4.3. Eligibility criteria

### 4.3.1.Inclusion criteria

- Participants should have experienced a coronary or cerebral event during 1 to 12 months before baseline. A coronary or cerebral event is defined as:
- Myocardial infarction *(validated and documented by a combination of clinical, enzymatic, ECG or coronarygraphics parameters);*
- Unstable angina *(validated and documented by a combination of clinical, enzymatic, ECG or coronarygraphics parameters);*
- A cerebral vascular accident *(defined by criteria validated in epidemiological studies).*
- The participants should be 45 - 80 years at baseline.

### 4.3.2. Exclusion criteria

- Age <45 years or >80 years;
- Cardiovascular pathology not well defined;
- Patients that are incapable of understanding the study protocol;
- Patients with a pathology that might interfere with homocysteine metabolism (*like use of methotrexate and antiepileptic drugs, hypothyroidism, psoriasis*, *etc.)*

## 4.4. Recruitment of patients

### 4.4.1. Eligibility judged by cardiologists and neurologists

The recruitment of subjects will be done through a network of cardiologists and neurologists, who voluntary have accepted to be correspondents of the study. The clinicians in this network will judge whether a patient is eligible for the study and they will facilitate the follow-up of the patients, either because they treat the patients themselves or they make it possible to contact the clinician in charge of the patient’s medical follow-up.

### 4.4.2. Responsibility of the cardiologists and neurologists

The clinicians have the responsibility to give potential study participants (i.e. patients that meet the eligibility criteria) the first information about the study protocol. They will explain that participation includes taking supplements daily, which will be provided to them. In addition, they will explain that 25% of all participants will receive a complete placebo treatment. The clinicians will also explain that despite the fact that they might receive only placebo supplements, all participants will benefit from the (free) annual physical and biological check-up that enables early identification of health problems as the results are given to the participants.

### 4.4.3. Screening questionnaire

The clinicians will offer a short questionnaire (1 page) to all patients willing to participate. The questionnaire will ask for 1) information by which inclusion of patients can be verified, 2) the patients address and 3) for permission to give the questionnaire’s information to the SU.FOL.OM3 team. Clinicians that have recruited patients will inform the SU.FOL.OM3 team by using an internet site, or by fax or mail (using a simple form designed for this use).

### 4.4.4. Recruitment period

The inclusion of all patients will take place over a period of 12 months.

# 5. Registration and randomization by su.fol.om3 team

## 5.1. Registration

Once the clinicians have obtained a completed questionnaire from potential participants, the SU.FOL.OM3 team will contact the patient in order to arrange an appointment. This appointment will take place in a local facility with a simply equipped laboratory, during which the SU.FOL.OM3 team can verify eligibility.

At this appointment the participants will receive a unique identification number and sign a informed consent form conform the recommendations of the local ethical committee (CCPPRB Hôpital Cochin). Once informed consent is given, a blood sample is taken.

## 5.2. Randomization

Patients will be randomly assigned to a treatment group by means of a computerized system. The randomization will be stratified to age, sex, and the type of pathologies and/or treatment. Once participants are randomized, they will receive their supplementation (enough for 1 year) in a package carrying their identification number.

# 6. Treatment

## 6.1. Treatment regimen and compliance

The participants will take the supplements orally, preferable one in the morning and one in the evening. The supplements are provided in a package containing 52 blisters of 2 x 7 capsules, each corresponding to one week of treatment. From experience with the SU.VI.MAX study (Hercberg et al, 1998) it is known that this type of blister package facilitates the consumption of the capsules, which enhances compliance of the participants.

## 6.2. Length of treatment

Participants will use supplements for 5 consecutive years. At the first appointment (registration and randomization) participants receive supplements for the first treatment year. The supplements for the following 4 years will be supplied each year at the annual physical and biological check-up of the participants.

## 6.3. Supplements and packaging

The supplements exist of soft gelatin capsules. These capsules either contain the B-vitamins, the omega n-3, or the placebo substance. The placebo capsules will look and taste identical to the active supplements.

The raw material for the omega-3 capsules will be provided by the laboratory Pierre Fabre. Manufactures of B-vitamins will provide the contents of the B-vitamin capsules. The gelatin capsules will be manufactured by RP Scherer. The realization of the capsules and their blister packaging will be done under the responsibility of the Laboratory Pierre Fabre.

# 7. Monitoring of the participants

## 7.1. Annual check-up

The clinical and biological monitoring includes an annual physical and biological check-up during which the participants are examined in a laboratory facility of their choice near to their home, e.g. a health examination center, a laboratory participating in the SU.FOL.OM3 study, or in the mobile units used for the SU.VI.MAX study (Hercberg et al, 1998) in those sites where local solutions can not be found.

## 7.2. Follow-up questionnaire for participants

Each year a simple follow-up questionnaire will be provided to the participants allowing to collect information on occurred health events (diseases, hospitalizations, etc.) and on the evolution of certain risk factors and lifestyle factors (smoking, dietary habits, physical activity, etc.).

## 7.3. Follow-up by clinicians

The clinicians who follow the SU.FOL.OM3 patients are requested to report (through internet, mail, fax or telephone) any relevant information on health-events of the SU.FOL.OM3 patients.

## 7.4. Biological parameters to be measured

In the blood that is drawn during the first appointment (baseline: T0) the following parameters will be determined:

- plasma homocysteine
- plasma vitamin B12
- plasma pyridoxal phosphate (circulating form of vitamin B6)
- plasma folate
- red blood cell folate
- 677 C>T polymorphism in the gene encoding for MTHFR (and possibly other polymorphism’s)
- fatty acid profile

Depending on the available means, markers of oxidative stress and antioxidant defenses, the lipid profile, platelet aggregation (test with thrombin and the AMP, possibly factor VIII, resistance to the protein C, fibrinogen, etc.) will also be measured.

Every year, for 5 subsequent years, around the date of baseline (T1, T2, T3, T4 and T5), the plasma homocysteine concentration will be measured. The plasma and erythrocyte concentration of folate and the fatty acids profiles will be measured at T2, T4 and T5. The plasma concentration of vitamin B12 and pyridoxal phosphate will be measured at T5.

A biobank will be set up during the first study year (12 straws of 500 µl of plasma and buffy-coat, for each subject).

## 7.5. Measurement of intermediate endpoints

Certain intermediate endpoints of cardiovascular disease will be measured on subsamples, i.e. hemostatic and endothelial cell markers. In participants living in the Parisian area the endothelial function will be measured as well as a detailed investigation of the carotid artery (intima media thickness, diameter, pulsatory waves, central pulse pressure, compliance, distensibility, calcifications) and also the existence of plaques and/or stenosis at different sites of the vessel system. Besides these cardiovascular disease intermediates the SU.FOL.OM3 study also plans to evaluate the effects on cognitive function. Several studies indicate that an optimal B-vitamin status may improve cognitive function (Selhub et al, 2000; Nilson et al, 2001), but a real effect never has been proven in a long-term intervention trial.

## 7.6. Hard endpoints

The primary endpoint is the *total number* of fatal and non-fatal events due to a

- myocardial infarction
- unstable angina
- ischemic cerebrovascular accidents

Secondary endpoints are:

- total mortality
- cardiovascular mortality
- myocardial infarctions
- unstable angina
- ischemic cerebral vascular accidents
- arteriopathies
- venous thromboses

## 7.7. Validation of endpoints

A coding form will be used to classify all the cardiovascular events. An independent committee of cardiologists and neurologists will be set up to ensure the validation of the events according to the form. Data of patients will be prepared by the SU.FOL.OM3 team and presented to this committee which has the responsibility to decide whether the collected information is sufficient enough to allow coding of the event. In case of insufficient information, additional information will be obtained before coding. After examination of the data, the committee will sign a dated card testifying for the encoding.

# 8. Statistical analyses

SU.FOL.OM3 has a factorial design, therefore the data will be analyzed by groups. Kaplan-Meier curves, which describe the proportion of participants that survived the period between the date of randomization and the date of censoring. The first event will be censored according to primary endpoints. Other ischemic events that occur during continuation of the study will be recorded as secondary endpoints.

The Kaplan-Meier curves will be compared by use of log-rank tests (analyses by intention to treat). Cox proportional hazard models will be used to test the effect of supplementation while adjusting for other variables. In addition interaction terms can be included and evaluated in these models. The data processing will be done by intention to treat.

# 9. Sources of bias and issues that may affect the power of the study

## 9.1. Unexpected unblinding of the protocol

The comparability of the treatment groups would be seriously affected when participants would find to which treatment group they are allocated. Therefore, it will be thoroughly checked whether the supplements are indistinguishable.

## 9.2. Compliance problems

The nutritional doses used are sufficient in such a way that if participants forget to take their supplements accidentally this will not have notable long term consequences.

## 9.3. Loss to follow-up

This happens when the endpoints of interest can not be established (except for mortality) or when subjects stop taking the supplements. Loss to follow-up implies the loss of power, and/or introduction of bias. Nevertheless, loss to follow-up is likely to occur independently of whether participants receive an active supplement or a placebo.

The percentage ‘lost to follow-up’ and ‘drop-out’ is estimated at 10 %, and this was taken into account in the power calculation. To reduce to the loss to follow-up to a minimum, a monitoring system and a very strict follow-up system will be set up. For subjects that wish to stop with the study (drop-out), a control system will be set up implicating annual contact with the subjects, contact with the clinicians who follow the patients, and ascertainment of the vital status of these patients.

# 10. Mobilization and motivation of participating health professionals

## 10.1. Mobilization of clinicians

The mobilization of the clinicians, who will be working in general hospitals (cardiologists, neurologists, services of internal medicine), independently, or in cardiovascular or neurovascular rehabilitation centers, will be done through various professional societies and colleges of specialists, in particular within the framework of the partnership with the National College of Cardiology. Potential members of the network will be informed about the goals and practical organization SU.FOL.OM3 through leaflets, booklets, meetings, in particular within the framework of the continuous training that specialists receive.

## 10.2. Motivation of clinicians and other health professionals

In order to stimulate the interest and to maintain the motivation of the participating clinicians and biochemists of the SU.FOL.OM3 network, the following is proposed:

- all the participating clinicians and other health professionals involved will be informed regularly on the status of the study and its principal results;
- all the participating clinicians and other health professionals involved will be quoted in scientific publications describing the methodology and the results of the study as members of the network;
- the members of the strategic committee of SU.FOL.OM3, the participating clinicians and the other health professionals will have the possibility of exploring the collective results of the study in local and regional scientific conferences (after agreement of national coordination);
- an internet site will be created especially for the SU.FOL.OM3 network (access to the network by means of a password). This will make it possible to circulate information on the study and to provide bibliographical data and scientific references to work published in this field (information on paper will be sent to those who prefer this over the internet);
- annual meetings will be organized for the participating health professionals to exchange information and to present available results (these meetings organized in Paris will be financed by the national coordination of the study);
- information will also be sent regularly to cardiologists and general practitioners who may have responsibility over patients participating in SU.FOL.OM3, but who are not affiliated to a general hospital. This information will include the justification of the study, its course and its state of advance;
- for the clinicians, depending on the financing obtained and available, a reward will be proposed in the form of a present (to be defined). The value of this present will be proportional to the number of subjects included by the clinicians.

# 11. Time schedule

Pilot: May-June 2002

Inclusion: October 2002-October 2003

Follow-up: October 2002-October 2008

# 12. References

| Albert CM et al (1998). Fish consumption and risk of sudden cardiac death. JAMA, 7: 23-28. |
| --- |
| Appel LJ et al (1993). Does supplementation of diet with fish oil reduce blood pressure ? Arch Intern Med, 153: 1429-1438. |
| Arnesen E et al (1995). Serum total homocysteine and coronary heart disease. Int J Epidemiol, 24: 704. |
| Ascherio A et al (1995). Dietary intake of marine n-3 fatty acids fish intake and the risk of coronary disease among men. NEJM, 332: 997-982. |
| Bang et al (1980). The composition of the Eskimo food in north western Greenland. Am J Clin Nutr, 33: 2657-2661. |
| Bjorkegren K and Svardsudd K (1999). Elevated serum levels of menthylmalonic acid and homocysteine in elderly people. A population-based intervention study. J Internal Med, 246: 317-324. |
| Bordin P et al (1998). Effects of fish oil supplementation on apolipoproin B100 production and lipoprotein metabolism in normolipidaemic males. Eur J Clin Nutr, 58: 104-109. |
| Bostom AG and Garber C (2000). Endpoints for homocysteine-lowering trials. Lancet, 355: 511-512. |
| Bots ML et al (1999). Homocysteine and short-term risk of myocardial infarction and stroke in the elderly. Arch Intern Med, 159: 38-44. |
| Boushey CJ et al (1995). A quantitative assessment of plasma homocysteine as a risk factor for vascular disease. Probable benefits of incresing folic acid intakes JAMA, 274: 1049-1057. |
| Brasttsrom LE et al (1988). Folic acid-an innocuous means to reduce plasma homocysteine. Scand J Clin Lab Invest, 48: 215-221. |
| Brouwer IA et al (1999). Dietary folate from vegetables and citrus fruit decreases plasma homocysteine concentrations in humans in an dietary controlled trial. J Nutr , 129: 1135-1139. |
| Brown AA and Hu FB (2001). Dietary modulation of endothelial function: implications for cardiovascuslar disease. Am J Clin Nutr, 73: 673-686. |
| Burr ML et al. Effect of changes in fat, fish, and fibre intakes on death and myocardial reinfarction : diet and reinfarction trial (DART). Lancet, ii: 757-761. |
| Clarke R and Armitage J (2000). Vitamin supplements and cardiovascular risk: review of the randomized trials of homocysteine-lowering vitamin supplements. Semin Thromb Hemost, 26: 341-348. |
| Connor WE (2000). Importance of n-3 Importance of n-3 fatty acids in health and disease. Int J Clin Nutr, 71: 171S-175S. |
| Dalery K et al (1995). Homocysteine and coronary artery disease in french Canadian subjects : relation with vitamins B12,B6, pyridoxal phosphate, and folate. Am J Cardiol, 75: 1107-11 |
| Danesh J and Lewington S (1998). Plasma homocysteine and coronary heart disease. J Cardiovasc Res, 5: 229-232. |
| de Bree A et al (2001). The association between B vitamin intake and plasma homocysteine concentration in the general Dutch population aged 20-65 years. Am J Clin Nutr, 73: 1027-1033. |
| De Jong SC et al (1999). Normohomocysteinaemia and vitamin-treated hyperhomocysteinaemia are associated with similar risks of cardiovascular events in patients with premature peripheral arterial occlusive disease. A prospective cohort study. J Intern Med, 246: 87-96. |
| De Lorgeril M et al (1999). Mediterranean diet, traditional risk factors, and the rate of cardiovascular complication after myocardial infarction : final report of the Lyon. Circulation, 99: 779-795. |
| Den Heijer M et al (1998). Vitamin supplementation reduces blood homocysteine levels. Arterioscler Throm Vasc Biol, 18: 356-361. |
| Dierkes J et al (1998). Folic acid and vitamin B6 supplementation and plasma homocysteine concentrations in healthy young women. Internat J Vit Nutr Res, 68: 98-103. |
| Du Vigneaud V et al (1933). Growth-promoting properties of homocystine when added to cystine-deficient diet and proof of structure of homocystine. J Biol Chem, 101: 719-726. |
| Dyerberg J and Bang HO (1982). A hypothesis on the development of acute myocardial infarction in Greenlanders. Scand J Clin Lab Invest Suppl, 161: 7-13. |
| Finkelstein JD (1990). Methionine metabolism in mammals. J Nutr Biochem, 1: 228-237. |
| Fohr IP et al (2002). 5,10-Methylenetetrahydrofolate reductase genotype determines the plasma homocysteine-lowering effect of supplementation with 5-methyltetrahydrofolate or folic acid in healthy young women. Am J Clin Nutr, 75: 275-282. |
| Frosst P et al (1995). A candidate genetic risk factor for vascular disease a common mutation in methylenetetrahydrofolate reductase. Nat Genet, 10: 111-13. |
| Genest JJ et al (1990). Plasma homocyst(e)ine concentrations in men with premature coronary artery disease. J Am Coll Cardiol, 16: 114-1119. |
| Gerster H et al (1993). Fish oil (n-3 long-chain PUFAS) and blood pressure. Nutrition, 17: 15-24. |
| Gester H (1995). The use of n-3 pufas (fish oil) in enteral nutrition. Internat J Vit Nutr Res, 53: 479-486. |
| Giles WH et al (1998). Serum folate and risk for coronary heart disease : results from a cohort of US adults. Ann Epidemiol, 8: 490-6. |
| Gissi-Prevenzione Investigators (1999). Dietary supplementation with n-3 polyunsaturated fatty acids and vitamin E after myocardial infarction: results of the GISSI-Prevenzione Trial. Lancet, 354: 447-455. |
| Gottlicher M et al (1992). Fatty acids activate a chimera of the clofibric acid-activated receptor and the glucocorticoid receptor. Proc Nath Acad Sci USA, 89: 4653-4657. |
| Graham IM et al (1997). Plasma homocysteine as a risk factor for vascular disease. The European Concerted Action Project. JAMA, 277: 1775-81. |
| Grynberg A et al (1997). Acides gras oméga-3 et prévention cardiovasculaire. Cah Nutr Diet, 32: 107-114. |
| Guize L and Richard JL (1990). De l’épidémiologie en cardiologie. Projections, la santé au futur 2: 127-134. |
| Hackam DG et al (2000). What level of plasma homocyst(e)ine should be treated? Effects of vitamin therapy on progression of carotid atherosclerosis in patients with homocyst(e)ine levels above and below 14 micromol/L. Am J Hypertens, 13: 105-110. |
| Haglund O et al (1998). Effects of fish oil alone and combined with long chain (n-6) fatty acids on some coronary risk factors in male subjects. J Nutr Biochem, 9: 629-635. |
| Hankey GH and Eikelboom JW (1999). Homocysteine and vascular disease. Lancet, 354: 407-413. |
| HCSP (Haut Comité de la Santé Publique) (2000). Pour une politique nutritionnelle de santé publique en France. Rapport du HCSP. Editions ENSP. |
| Hercberg S. et al (1998). A primary prevention trila using nutritional doses of antioxidant vitamins and minerals in cardiovascular diseases and cancers in a general population. The SU.VI.MAX study – design, methods, and participants characteristics. Control Clin Trials, 19: 336-351. |
| Hill C et al (1996). Analyse statistique des données de survie. D. Schwartz Eds. INSERM, Médecine –Sciences / Flammarion. |
| Kang J et al (1996). Antiarrhythmic effects of polyunsatured fatty acids. Recent studies. Circulation, 94: 1174-1780. |
| Kang S et al (1986). Proteine-bound homocyst(e)ine. J Clin Invest, 77: 1482-1486. |
| Kark JD et al (1999). Nonfasting plasma total homocysteine level and mortality in middle-aged and elderly men and women in Jerusalem. Ann Intern Med, 131: 321-330. |
| Keller H et al (1993). Fatty acids and retinoids control lipid metabolism through activation of peroxisome proliferator-activated receptor-retinoid X receptor heterodimers. Proc Nat Acad Sci USA, 90: 2160-2164. |
| Kelly et al (1997). Unmetabolized folic acid in serum: acute studies in subjects consuming fortified food and supplements. Am J Clin Nutr, 65: 1790-1795; |
| Knapp HR (1989). Omega-3 fatty acids, endogenous prostaglandins and blood pressure regulation in humans. Nutr Rev, 47: 301-313. |
| Knapp HR (1990). Polyunsaturates, endogenous eicosanoids, and cardiovascular disease. J Am Coll Nutr, 9: 344-351. |
| Krämer HJ et al (1996). Fish oil fatty acids and human platelets : dose-dependant decrease in dienoic and decrease in trienoic thromboxane generation. Biochem Pharmacol, 52: 1211-1217. |
| Kromhout D (1985). The inverse relation between fish consumption and 20 years mortality from coronary disease. NEJM, 312: 1205-1209. |
| Landgren F et al (1995). Plasma homocysteine in acute myocardial infarction : homocystein-lowering effect of folic acid. J Intern Med, 237: 381-388. |
| Lemaitre D et al (1997). Effects of fatty acids on human platelet glutathione peroxidase : possible role of oxidative stress. Biochem Pharmacol, 53: 479-486. |
| Lu G et al (1999). Omega-3 fatty acids after lipoprotein subfraction distributions and the in vitro conversion of very low density lipoproteins to low density lipoproteins. J Nutr Biochem, 10: 151-158. |
| Malinow MR (1990). Hyperhomocysteinemia : a common and easily reversible risk for occlusive atherosclerosis. Circulation, 81: 2004-6. |
| Malinow MR et al (1996). Plasma homocyst(e)ine levels and graded risk for myocardial infarction : findings in two populations at contrasting risk for coronary heart disease. Atherosclerosis, 126: 27-34. |
| Mc Cully KS (1969). Vascular pathology of homocysteinemia : implications for the pathogenesis of arteriosclerosis. Am J Pathol, 56: 111-128. |
| McDowell IF and Lang D (2000). Homocysteine and endothelial dysfunction: a link with cardiovascular disease. J Nutr, 130: 369S-372S. |
| Morris MC et al (1992). Does fish oil lower blood pressure ? A meta-analysis of controlled trials. Circulation, 88: 523-533. |
| Nair SS et al (1997). Prevention of cardiac arrythmia by dietary (n-3) polyunsatured fatty acids and their mechanism of action. J Nutr, 127: 383-393. |
| Naurath HJ et al (1995). Effects of vitamin B12 Folate and vitamin B6 supplements in elderly people with normal serum vitamin concentrations. Lancet, 346: 85-89. |
| Nenseter MS et al (1992). Effect of dietary supplementation with n-3 polyunsatured fatty acids on physical properties and metabolism of low density lipoprotein in humans. Atheroscler Thromb, 12: 369-379. |
| Nilsson K et al (2001). Improvement of cognitive functions after cobalamin/folate supplementation in the elderly patient with dementia and elevated homocysteine. Int J Geriatr Psychiatry, 16: 609-614. |
| Nygard O et al (1997). Plasma homocysteine levels and mortality in patients with coronary artery disease. N Engl J Med, 337: 230-236. |
| Nygard O et al (1999). Plasma homocysteine and cardiovascular disease. J Intern Med, 246: 425-454. |
| Oomen et al (2000). Fish consumption and coronary heart disease mortality in Finland, Italy and the Netherlands. Am J Epidemiol, 151: 999-1006 |
| Pancharuniti N et al (1994). Plasma homocyst(e)ine, folate and vitamin B-12 concentrations and risk for early-onset coronary artery disease. Am J Clin Nutr, 59: 940-948. |
| Passmore R and Eastwood MA (1986). Fats. In: Davidson, Passmore, eds. Human nutrition and dietetics. Edinburg: Churchill Livingstone: 54-69. |
| Rasmussen K et al (1996). Age-and gender-specific intervals for total homocysteine and methylmalonic acid in plasma before and after vitamin supplementation. Clin Chem, 42: 630-636. |
| Refsum H et al (1998). Homocysteine and cardiovascular disease. Annu Rev Med, 49: 31-62. |
| Riddell LJ et al (2000). Dietary strategies for lowering homocysteine concentrations. Am J Clin Nutr, 71: 1448-1454. |
| Roquebrune JP (1994). Le livre de la prévention cardio-vasculaire. Fédération française de cardiologie. |
| Santhosh-Kumar CR et al (1997). Unpredictable intra-individual variations in serum homocysteine levels on folic acid supplementation. Eur J Clin Nutr, 51: 188-192. |
| Schnyder G et al (2001). Decreased rate of coronary restenosis after lowering of plasma homocysteine levels. NEJM, 345: 1593-1600. |
| Segal-Isaacson CJ and Wylie-Rosett J (1999). The cardiovascular effects of fish oils and omega-3 fatty acids. Heart Dis, 1: 149-54. |
| Selhub J et al (1993). Vitamin status and intake as primary determinants of homocysteinemia in an elderly population. JAMA, 270: 2693-2698. |
| Selhub J, et al (2000). B vitamins, homocysteine, and neurocognitive function in the elderly. Am J Clin Nutr, 71: 614S-620S. |
| Siscovick D et al (1995). Dietary intake and cell membrane levels of long chain n-3 polyunsatured fatty acids and the risk of primary cardiac arrest. JAMA, 1: 1363-1367. |
| Stampfer MI et al (1992). A prospective study of plasma homocysteine and risk of myocardial infarction in US physicians. JAMA, 268: 877-81. |
| Stehouwer CDA et al (1998). Serum homocysteine and risk of coronary heart disease and cerebrovascular disease in elderly men : a 10-year follow-up. Arterioscler Thromb Vasc Biol, 18: 1895-1901. |
| Stroes ES et al (2000). Folic acid reverts dysfunction of endothelial nitric oxide synthase. Circ Res, 86: 1129-1134. |
| Thambyrajah J and Townend JN (2000). Homocysteine and atherothrombosis - mechanisms for injury. Eur Heart J, 21: 967-974. |
| Ubbink et al (1991). The prevalence of homocysteinemia and hypercholesterolemia in angiographically defined coronary heart disease. Klin Wochenschr, 69: 527-534. |
| Ubbink JB et al (1993). Hyperhomocysteinemia and the response to vitamin supplementation. Clin Invest. 1993, 71: 993-998. |
| Ueland PM and Refsum H (1989). Plasma homocysteine, a risk factor for vascular disease: plasma levels in health, disease and drug theraphy. J Lab Clin Med 114: 473-501. |
| Ueland PM et al (2000). The controversy over homocysteine and cardiovascular risk. Am J Clin Nutr, 72: 324-332. |
| Verhaar MC et al (1998). 5-methyltetrahydrofolate, the active form of folic acid, restores endothelial function in familial hypercholesterolemia. Circulation, 97: 237-241. |
| Vermeulen EG et al (2000a). Normohomocysteinaemia and vitamin-treated hyperhomocysteinaemia are associated with similar risks of cardiovascular events in patients with premature atherothrombotic cerebrovascular disease. A prospective cohort study. Neth J Med, 56: 138-146. |
| Vermeulen EG et al (2000b). Effect of homocysteine-lowering treatment with folic acid plus vitamin B6 on progression of subclinical atherosclerosis : a randomized, placebo-controlled trial. Lancet, 355: 517-522. |
| Vollset SE et al (1985). Fish consumption and mortality from coronary heart disease. NEJM, 313: 820-821. |
| Wald NJ et al (1998). Homocysteine and ischemic heart disease. Results of a prospective study with implications regarding prevention. Arch Intern Med, 158: 862. |
| Woodside JV et al (1998). Effet of B-goup vitamins and antioxidant vitamins on hyperhomocysteinemia : a double-blind, randomized, factorial-design, controlled trial. Am J Clin Nutr, 67: 858-866. |
| Wu LL et al (1994). Plasma homocyst(e)ine as a risk factor for early familial coronary artery disease. Clin Chem, 40: 552-561. |
